# CT colonography: revisited after 30 years

**DOI:** 10.1186/s13244-025-02038-x

**Published:** 2025-07-31

**Authors:** Mehdi Cadi, Charles André Cuenod, Philippe A. Grenier, Aurélien Saltel, Mohamed Abdulbaki, Gilles Manceau, Mehdi Karoui

**Affiliations:** 1 Department of Radiology, Paris Cité University, Assistance Publique Hôpitaux de Paris, Georges Pompidou University Hospital, Paris, France; 2 Radiologie Paris Ouest - Imagerie Cardinet, Neuilly-sur-Seine, France; 3 Department of Clinical Research and Innovation Hôpital Foch, Suresnes, France; 4 Department of Digestive and Oncological Surgery, Paris Cité University, Assistance Publique–Hôpitaux de Paris, Georges Pompidou University Hospital, Paris, France

**Keywords:** CT colonography, Laparoscopic surgery, Cancer screening, Diverticular disease, Incomplete colonoscopy

## Abstract

**Abstract:**

Computed tomography colonography (CTC), also known as virtual colonoscopy, is a well-tolerated, minimally invasive and effective procedure. Used for over two decades and supported by extensive studies and meta-analyses, CTC has demonstrated performance comparable to that of optical colonoscopy (OC). However, CTC remains generally underutilized in many countries, including the United States of America; in contrast, in some countries, such as the United Kingdom, it is widely used.

CTC requires bowel preparation with laxative and fecal contrast-agent tagging, followed by colonic distension with low-pressure, automated, CO_2_ insufflation. It enables detailed image analysis with postprocessing software and is highly sensitive and specific for detecting cancers and significant benign precursors ≥ 10 mm (adenomatous and sessile-serrated polyps) years before potential malignant transformation.

After reviewing the state of the art of CTC acquisition, analysis and reporting, we wrote this article to update the new, potential and emerging CTC indications. CTC is increasingly used after incomplete OC, for undetermined colonic anomalies, in elderly and/or fragile patients or when OC is refused. Recent routine clinical use has broadened CTC’s applications, proving its usefulness in local colon-cancer staging, preoperative laparoscopic surgery planning, and selecting patients with severe diverticular disease for elective sigmoidectomy.

**Critical relevance statement:**

Beyond its excellent performance in detecting advanced adenomas and cancers, CTC provides precise staging of locally advanced tumors, guiding decisions on neoadjuvant therapy, and coupled with contrast-enhanced thoracic–abdominal–pelvic acquisition, enables comprehensive, preoperative evaluation for laparoscopic colectomy.

**Key Points:**

- CT colonography (CTC) and optical colonoscopy (OC) are similarly able to detect advanced adenomas (≥ 10 mm) and early-stage colorectal cancer.
- CTC enables a “one-stop shop” examination for laparoscopic surgery planning, with precise localization and detailed vascular mesenteric mapping.
- With the rise of neoadjuvant treatments for advanced colorectal cancer, CTC may become pivotal in radiological staging.

**Graphical Abstract:**

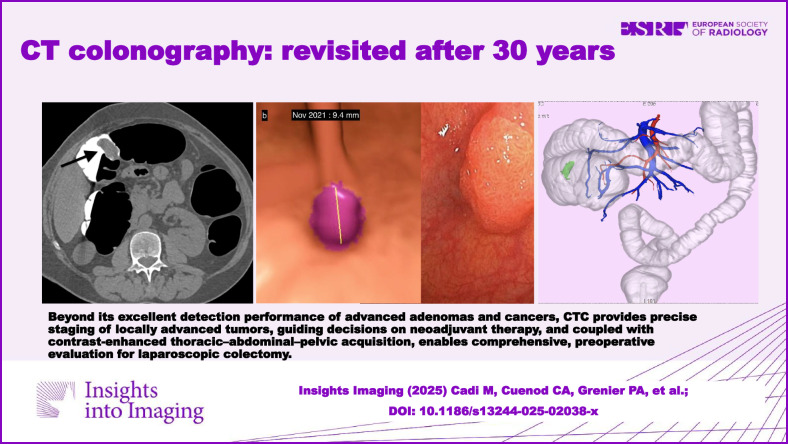

## Introduction

Computed tomography colonography (CTC) is a well-tolerated and complication-free diagnostic procedure. Since the first description in 1994 by Vining et al [1], this technique has been available for over 30 years.

It has been extensively evaluated in asymptomatic and symptomatic individuals through numerous studies [2–7] and meta-analyses [8, 9]. The procedure requires colon preparation with laxatives and oral contrast-agent tagging [10]. The colon is distended using a low-pressure CO_2_ insufflator that enables detailed analysis with advanced postprocessing imaging software. Beyond its excellent sensitivity and specificity for detecting cancers, CTC is able to identify significant benign precursors (≥ 10 mm), such as adenomatous and sessile-serrated polyps, years before they may transform into cancer [11, 12].

Despite its proven efficacy, CTC remains underutilized and/or almost nonexistent in some countries, contrasting sharply with others in Europe, such as the UK, Ireland and Italy. In the UK, for example, more than 120,000 CTC examinations, both in symptomatic and asymptomatic patients, were performed yearly, with some centers doing more than 3000 exams per year [13]. This underutilization is compounded by a workload that is continuously on the rise, the lack of enthusiasm among radiologists and biased messaging in the literature leading to ignore this important exam.

In recent years, routine clinical use of CTC has expanded its application fields. CTC has proven particularly beneficial in evaluating the local extension of colorectal cancer and preoperative planning via laparoscopy for colonic tumors [14–16].

In this review, we have consolidated extensive data and recent CTC advancements, reasserting its efficacy and broadening its clinical application, and highlighting the usefulness of CTC in preoperative planning and early detection of significant benign and malignant lesions.

## Technique

### Bowel preparation

The European Society of Gastrointestinal and Abdominal Radiology (ESGAR) recommends bowel preparation with laxatives and oral contrast agents for fecal tagging [10]. This technique is reproducible, effective and well-standardized, provided it is performed rigorously. It combines three objectives: achieving the cleanest possible colon, reducing the amount of liquid residue, and tagging of residual liquid and solid stools. This protocol stipulates that patients undergo bowel preparation the day before the examination, which comprises a liquid diet and the ingestion of either magnesium-citrate laxative or phosphosoda. Fecal tagging with oral barium or hyperosmolar/iso-osmolar iodine solutions or both is now considered mandatory for CTC [10].

**Fig. 1:**
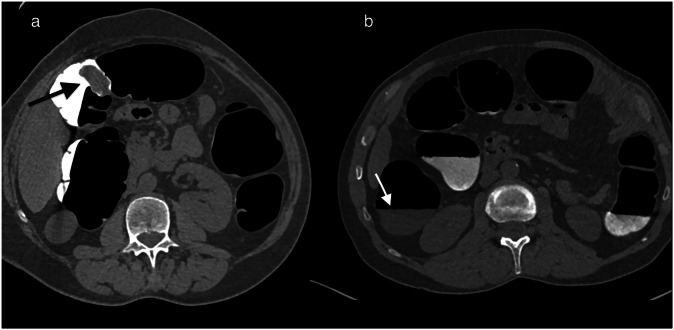
Optimal colon preparation for CT detection of colonic tumors. **a** Patient with state-of-the-art bowel preparation. The axial image shows the colonic lesion (indicated by the black arrow), exhibiting a tissue density that is easily detectable within an optimal radiological environment characterized by air density (< 1000 HU) and fluid tagging (> 1000 HU). **b** Another patient with incomplete fecal tagging. The axial slice reveals residual stool not marked by the oral contrast (white arrow), making it challenging to detect a colonic tumor

Our protocol includes a dual contrast-tagging regimen consisting of oral administration of diluted 5% barium sulfate (500 mL; Micropaque scanner®; Guerbet) and diatrizoate meglumine (50 mL; Telebrix Gastro®; Guerbet). That tagging regimen facilitates the detection of tissue lesions (50 HU) immersed in tagged residue (700 HU) (Fig. 1). It enables the detection of flat lesions covered by a thin layer of contrast agent, but it is difficult to specify which agent is involved in this mechanism. For patients with renal or cardiac insufficiency, polyethylene glycol Macrogol® can replace traditional laxatives, with 2 liters ingested the day before the examination, coupled with dual tagging. For elderly and/or frail patients, for whom the primary concern is detecting cancers or large polyps rather than small polyps, the preparation can be simplified. A 3-day low-residue diet is complemented by ingesting 50 mL of iodinated contrast agent the evening before the examination. The mini-laxative action of the hyperosmolar contrast agent completes the bowel preparation while ensuring stool tagging.

After an incomplete optical colonoscopy (OC), CTC can be done the same day if local conditions permit. This strategy spares the patient the inconvenience of undergoing a second bowel preparation. Avoiding wet-preparation liquid residues in OCs, stool tagging with an iodinated contrast agent is achieved by ingesting 30 mL of Telebrix Gastro® 2–3 h before the CT scan. If the first acquisition reveals incomplete stool tagging, it is preferable to postpone the examination to the following day to ensure homogeneous stool tagging. However, in accordance with ESGAR recommendations, when the patient has undergone an endoscopic procedure, especially when polypectomy and mucosectomy were performed, it is important to delay CTC by 2–6 weeks to avoid any theoretical risk of perforation during CO_2_ insufflation.

## Examination

Colon distension requires special attention to achieve a flawless level of quality. The examination’s quality directly impacts CTC performance, regardless of the reader’s expertise. Automatic low-pressure (15–20 mm Hg) CO_2_ insufflation is noninvasive, safe, well-tolerated and more comfortable for the patient than room air distension, because as CO_2_ is rapidly absorbed across the colonic mucosa (100× faster than room air) and is then expelled through the respiratory system. Active CO_2_ insufflation during image acquisition is needed to maintain optimal distention.

**Fig. 2:**
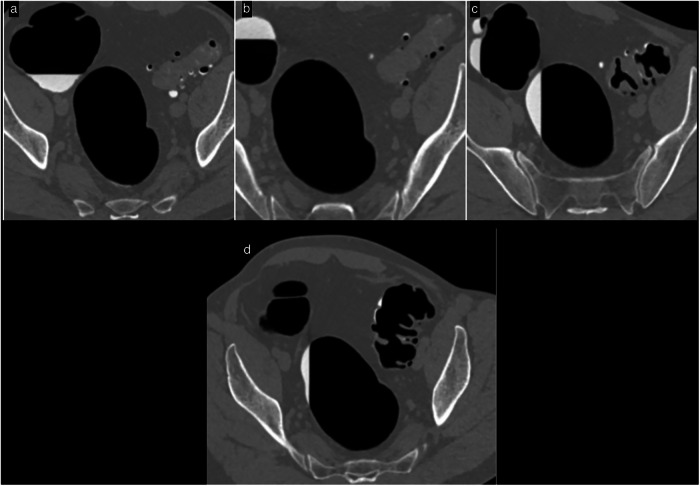
Patient referred for CTC because of an incomplete colonoscopy due to a fixed sigmoid colon. **a** Supine and **b** prone positioning did not allow colon distension of the diverticular sigmoid, despite a successful third acquisition (**c**) in the right lateral decubitus position. In that same position, colon distension was achieved only after intravenous glucagon (1 mg) administration instead of Buscopan not marketed in France (**d**)

Generally, CT acquisition combines supine and prone positions to mobilize residual stool. If the distension is inadequate, a third acquisition in the left lateral decubitus position may be necessary. In some cases, we use a spasmolytic agent such as an IV injection of 1 mg of Glucagon as a replacement for Butyl Scopolamine which is no longer marketed in France [17] (Fig. 2). When prone positioning is not feasible (e.g., for elderly patients, those with stomas, obesity, etc.), two acquisitions are obtained in the lateral decubitus positions. The scout view helps assess the quality of colon distension before the acquisitions. For symptomatic patients, a combination of supine and right lateral decubitus positioning improves the distension of the often suboptimally distended sigmoid and left colon.

A multi-detector CT scanner with at least 16 rows is sufficient for this examination, with ≤ 1.2-mm collimation and ≤ 0.7-mm slice interval. The acquisition is performed using a low-dose protocol (120 kV and ≤ 50 mAs). Dose modulation with iterative reconstructions achieves a total effective dose of 3 mSv, without compromising image quality.

In general, CTC does not require the injection of contrast material. However, contrast injection may become necessary in some circumstances, such as the detection of a cancerous lesion, the presence of highly pathological extracolonic abnormalities or colon cancer staging. A one-stop shopping variant, combining CTC with thoracic–abdominal–pelvic CT (TAP-CT) acquisition, provides a comprehensive evaluation in a single examination, including local tumor staging, mesenteric vascular mapping for surgical planning and distant metastasis assessment.

## Image analysis

Colonic lesion evaluation should be performed with both 2D and 3D imaging using a dedicated software allowing simultaneous analyses of 3D and 2D images. In practice, 3D viewing, being more sensitive than 2D for polyp detection, is used as a first approach, whereas 2D viewing is mostly used to confirm the soft-tissue nature of the detected polyp. In a 3D-reconstructed colon, antegrade and retrograde endoluminal fly-through navigation passes from the rectum to the cecum for each acquisition. In 2D mode, the images are reviewed by scrolling through the colon lumen, progressing from the rectum to the cecum, while combining multiplanar views. Wide-display window width and level settings, such as 2000 HU/0 HU, 1500 HU/–400 HU, or 1500 HU/–200 HU, are used to maximize polyp visualization.

**Fig. 3:**
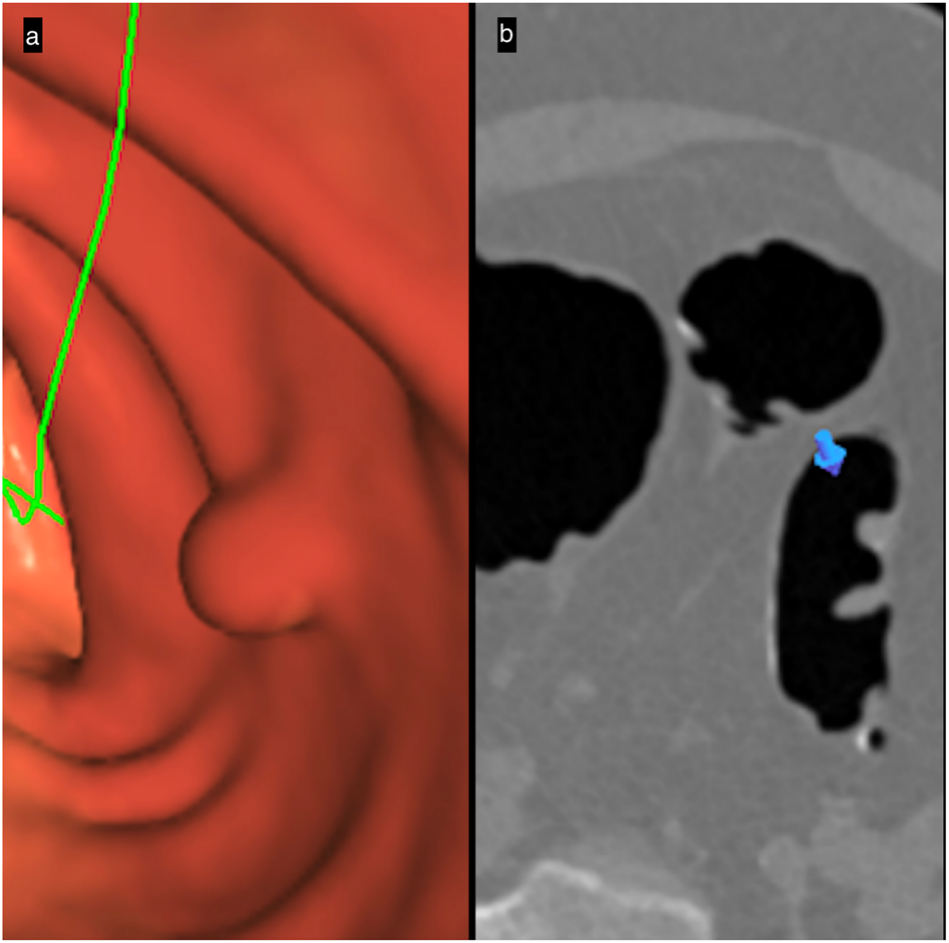
Primary 3D polyp detection: 3D–2D interaction. **a** 3D-endoscopic view makes detecting a candidate sessile polyp easier. Many workstations can show the axial slice corresponding to an abnormality seen in a 3D region of interest, but not the precise location on the 2D image. This functionality shortens interpretation time by avoiding the search for correspondences and provides an improved level of certainty about the nature of the detected anomaly. **b** The 2D-axial image confirms the polyp’s soft-tissue value, bookmarking on the 2D image (blue arrow) the specific location shown on the 3D image. Note that this bookmark is close to a fold and might appear only fleetingly during the scroll, making it easy to differentiate. The green center line allows fly-through navigation

To be more effective, the 3D approach needs dedicated software with specific features, such as effective interaction between 3D and 2D images. Currently, most workstations can easily show what the area detected in 2D mode looks like in 3D. However, when an abnormality is seen in 3D, many workstations can show the axial slice that corresponds to the identified 3D region of interest, but not the precise location on the 2D image that the 3D imaging is showing (Fig. 3). This simple functionality prevents prolonging interpretation time by avoiding the search for correspondences and achieves an improved level of certainty about the nature of the detected anomaly [18]. Manufacturers also provide computer-aided diagnosis tools, which, when used as a second reader, help reduce perceptual errors in detecting small (6–9 mm) polyps, even for expert radiologists.

To shorten the time required for image interpretation, an alternative 3D-display method is proposed with a panoramic view, such as “filet,” “dissection” or “unfolded cube” modes. However, the distortions generated by these techniques can create misleading images and ultimately slow down the interpretation process. The traditional 3D-endoscopic navigation mode has been the most widely used and published, but panoramic view performance has not been demonstrated with large trials.

Polyp size is a crucial factor in patient management, with the longest axis defining the size. It must be measured on the 2D multiplanar view in wide colon windows that provide the largest dimension. This linear measurement serves as a common reference for radiologists and gastroenterologists. Polyp measurement obtained on the 3D-endoluminal view correlates well with histopathological observations [12]. It is more accurate than the 2D measurement and is an ideal tool for monitoring polyp progression.

## CTC-detected anomalies

### Polypoid lesions

All potential polyps identified with 2D and 3D methods are subsequently assessed based on two criteria: a stable location relative to the colonic mucosa and the presence of a soft-tissue core. The presence of a fatty component suggests diagnoses like lipoma, fibrolipoma, or inverted diverticulum, which can be easily identified on a CT scan. Polyps can be sessile, pedunculated or flat. Colonic masses, including lateral spreading tumors (LSTs), also known as carpet lesions, are defined as tissue lesions > 3 cm. The likelihood of cancer or advanced histology within a polyp is strongly associated with its size.

### Subcentimeter polyps (≤ 9 mm)

These polyps are subdivided into diminutive polyps ≤ 5 mm and small polyps ≥ 6 mm. The former are typically hyperplastic or tubular adenomas and are always benign upon histological examination. American College of Radiology and ESGAR CTC guidelines do not mandate reporting these CTC. Non-reporting of diminutive polyps has been found to be cost-effective and safe. Hence, the threshold value to consider for reporting a polyp is ≥ 6 mm.

According to a study [19] on 43,000 subcentimeter polyps, the rate of advanced adenomas was 2.1% for polyps ≤ 5 mm and 5.6% for polyps 6–9 mm, and no cancer was detected. Based on those findings, the authors proposed: (1) the OC “resect-and-discard” strategy for subcentimeter polyps without histological analysis, (2) CTC surveillance at 3 years or polypectomy for polyps 6–9 mm, and (3) the uselessness of reporting polyps ≤ 5 mm. Pooler et al investigated predictive signs for histologically identified advanced-grade lesions (villous component, high-grade dysplasia, cancer) within small, 6–9-mm polyps based on longitudinal follow-up of 639 polyps in 475 subjects over a 16-year period [12]. Patients with those polyps underwent at least two successive follow-up CTCs every 3 years. Pertinently, 75% of those small polyps (*n* = 241) were either stable, diminished or resolved. Only 41 (6.4%) polyps were found to be histopathologically advanced (adenocarcinoma, high-grade dysplasia or villous content), including two cancers and 38 tubulovillous adenomas. The annual rate of polyp growth enabled histological stratification: < 33% for benign adenomas (tubular adenoma without dysplasia), ≥ 178% for advanced adenomas and ≥ 753% for the two cancers. Those results confirmed that the immediate risk of cancer for subcentimeter polyps is extremely low. CTC is not only a reliable tool for detecting and monitoring intermediate polyps but also for determining when to initiate therapeutic polypectomy. Being able to anticipate which polyps pose a significant threat could prevent numerous patients from undergoing unnecessary polypectomy, thereby avoiding the accompanying risks and expenses.

### Polyps ≥ 10 mm

The results of large single- and multicenter studies have clearly demonstrated that CTC’s ability to detect colorectal cancers and advanced adenomas (≥ 10 mm) is comparable to that of OC [8, 9, 20], with 90–94% sensitivity and 94–96% specificity.

## Non-polypoid lesions

Non-polypoid colorectal neoplasms are more likely to contain carcinoma than polypoid lesions, regardless of their size. Carcinoma-containing lesions are smaller in diameter than polypoid anomalies [21]. Non-polypoid lesions, i.e., rare and defined as slightly elevated lesions ≤ 3 mm high, comprise three subgroups: (1) flat polyps, (2) serrated polyps, and (3) LSTs.

### Flat polyps

Flat polyps are adenomas, defined as slightly elevated tissue lesions, ≤ 3 mm high, relative to the healthy mucosa. Those with a depressed center have the highest risk of being a carcinoma [21].

### Serrated polyps

These polyps are rare (3% of polyps), accounting for 15–20% of sporadic, non-familial colorectal cancers and have genetic, molecular and histological characteristics distinct from those of classic adenomas. They frequently arise in the proximal colon and usually have a diameter ≥ 10 mm at detection. They comprise three subcategories: hyperplastic, sessile and flat-serrated polyps.

*Hyperplastic-serrated polyps* are the most frequent (> 70%). They preferentially develop in the rectum and sigmoid colon and have no potential for malignancy. Their soft consistency, deformed by colonic insufflation, makes OC and CTC detection difficult.

**Fig. 4:**
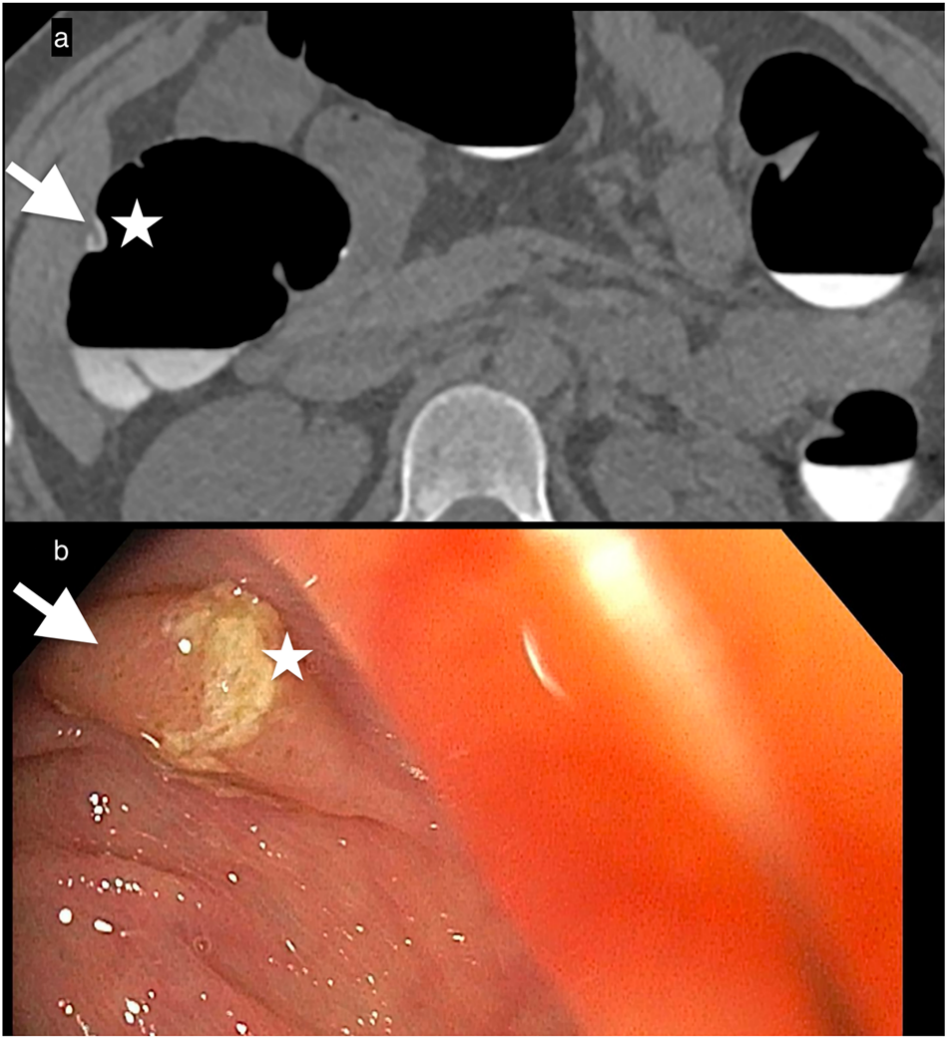
Sessile-serrated polyp (**a**) (7-mm) (white arrow) in the right colon on an axial image covered by a layer of oral contrast (star), while the remainder of the colon is clean, thereby facilitating its detection. **b** Endoscopic appearance of the same polyp (white arrow) shows a texture consistent with the surrounding healthy mucosa, and the yellowish stool covering it (star) draws the operator’s attention to the possible presence of a polyp

*Sessile-serrated polyps*, also known as sessile-serrated adenomas, are large polyps > 10 mm that form in the right colon and account for approximately one-fourth of all sporadic colorectal cancers. These lesions are often flat, only minimally elevated from the colon surface. Despite their minimally raised profile, the phenomenon of lesional contrast-material coating makes it possible for CTC to detect these lesions. That coating highlights the subtle morphological changes and further supports confidence that a true lesion exists despite its flat morphology (Fig. 4).

**Fig. 5:**
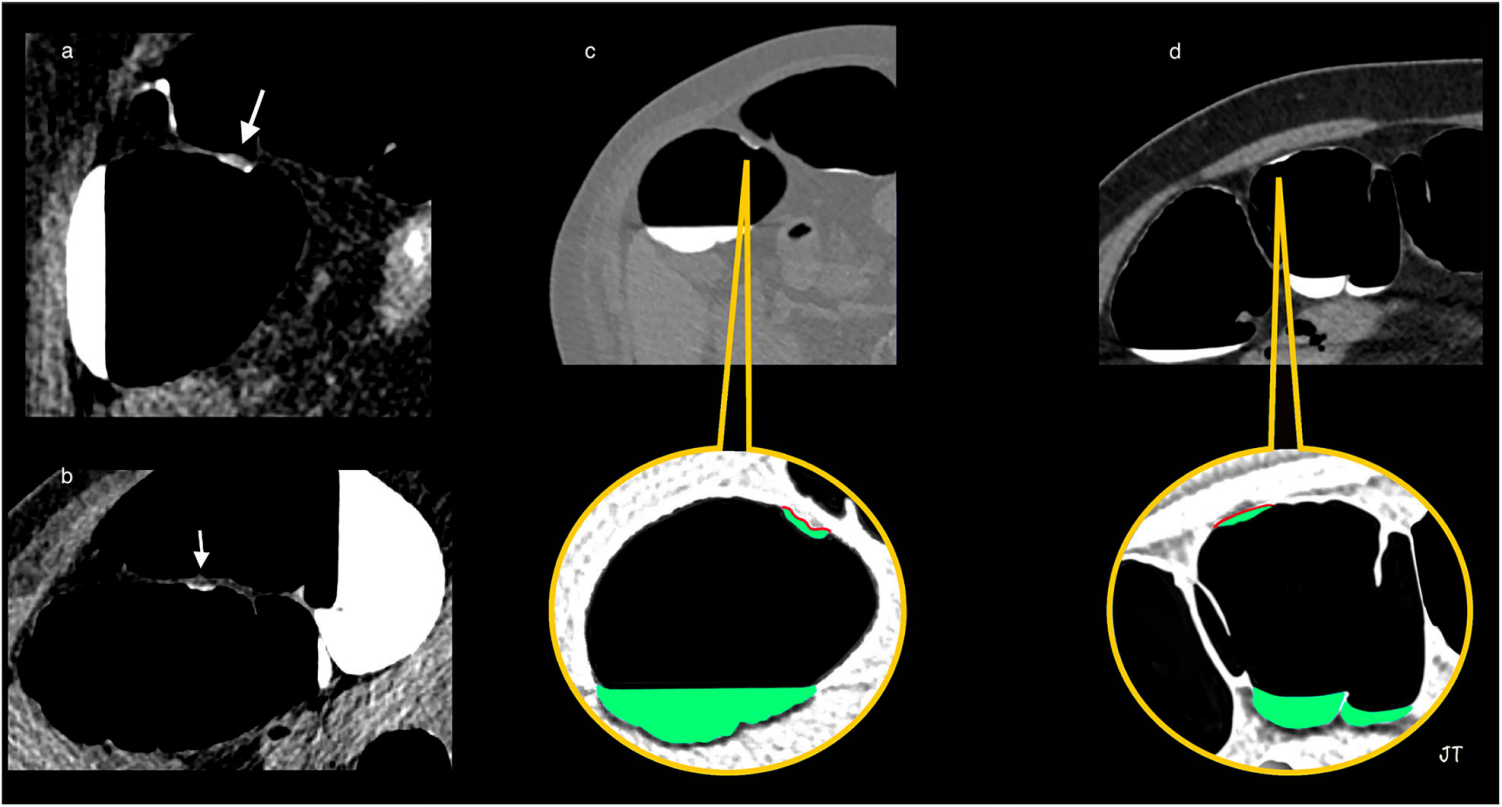
Flat-serrated lesions. This true flat polyp appears as a < 3-mm-high non-polypoid tissue lesion spreading on the surface at a fixed location on **a** right lateral decubitus and **b** left decubitus positioning, and covered by a thin film of oral contrast, demonstrating the floating oral contrast-coating sign. **c** The surface of the oral-contrast sign in contact with the polyp is depicted as an undulating red line. The green is an artifice to color liquid in the colon to improve visualization. **d** The stool adhering to the colon wall in another patient appears as the contrast-agent sign in contact with the wall and is seen as a smooth and regular surface with a concave appearance (red line)

*Flat-serrated polyps* appear the same as adjacent healthy mucosa on OC. That characteristic, along with their flat morphology, makes them particularly difficult to detect during OC. They are considered the main origin of interval colorectal cancers [20]. OC and CTC advances have improved their detection by exploiting their inherent secretion of a mucoprotein that forms a thin overlying film. CTC visualizes that thin layer of oral contrast agent as the “oral contrast-coating sign,” which has been observed in up to 86% of these patients [21, 22] (Fig. 5).

## LSTs or carpet lesions

**Fig. 6:**
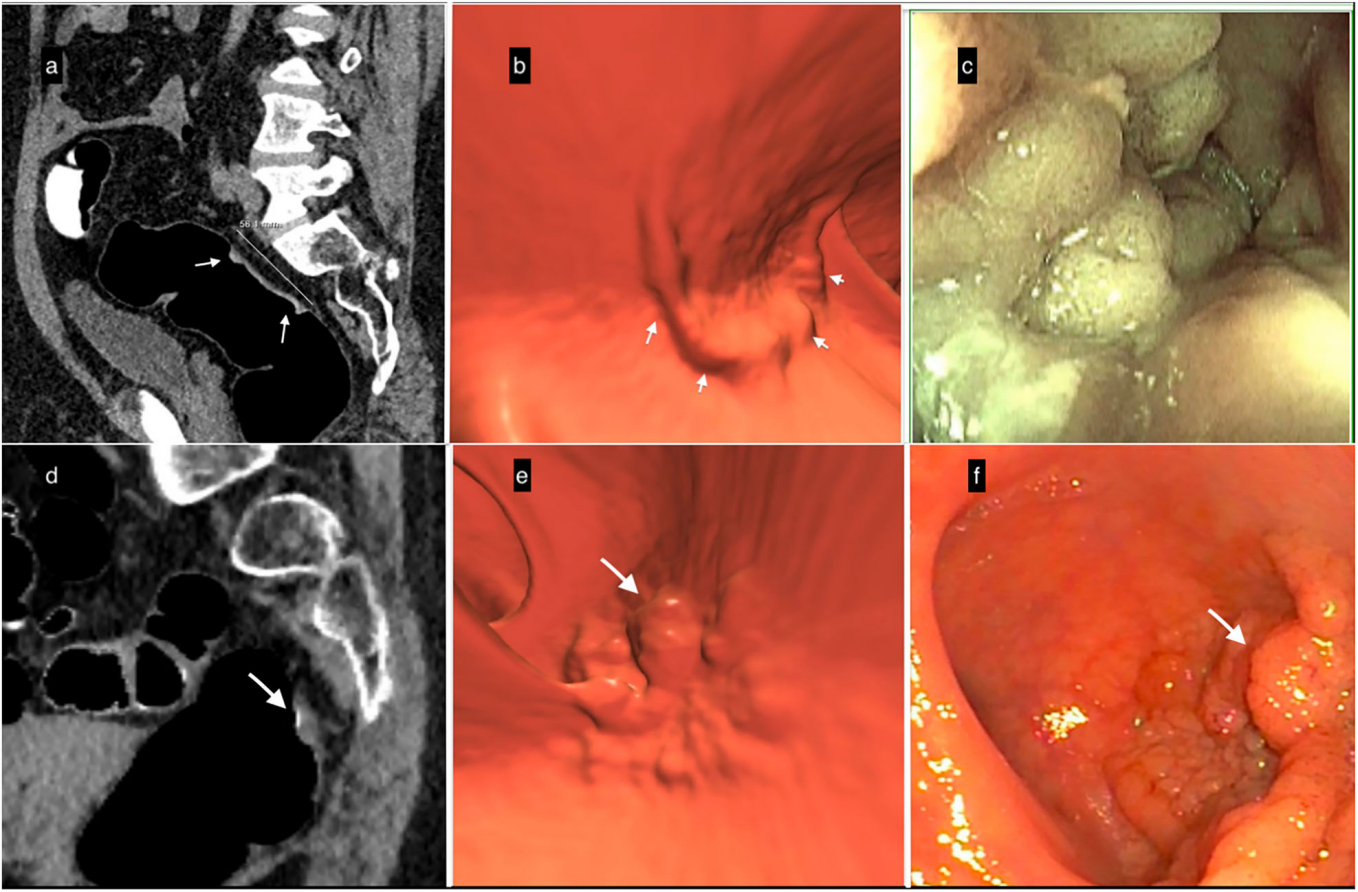
Lateral spreading tumors (LSTs). **a**–**c** Example of LST-non-granular (LST-NG). **a** Sagittal 2D-image with 40–400-HU abdominal window setting indicates a large 5.6-cm lesion with subtle diffuse thickening of the rectal wall (white arrows); the surface is partially covered by a thin layer of orally administered contrast agent. **b** The appearance of the villous mat on the 3D endoscopic view highlights the nodular aspect (white arrows) at the periphery of the lesion. **c** The optical colonoscopy view with the final diagnosis of a carpet villous adenoma with low-grade dysplasia. **d**–**f** Example of an LST-granular (LST-G) lesion. **d** Sagittal 2D image indicating nodular thickening of the rectum wall with a 10-mm nodule (white arrow). **e** The 3D endoscopic view visualized the irregular LST with a large nodule (white arrow), similar to **f** the endoscopic image obtained the same day after CTC. It confirmed the diagnosis and allowed mucosectomy with a final pathology report of a tubulous lesion with high-grade dysplasia

These rare tumors are seen as flat masses, usually detected when ≥ 3 cm in diameter and ≤ 2.5 mm thick. They are subdivided into granular lesions (LST-G), which have a polypoid appearance and sometimes contain a 10-mm macronodule, and non-granular lesions (LST-NG) (Fig. 6). They frequently develop near the rectum or the cecum. CTC visualizes them as flat, slightly elevated, surface lesions highlighted by the oral contrast-coating sign. CTC sensitivity to detect LSTs is high, close to 86% [23].

## Extracolonic abnormalities

While the primary objective of CTC is the detection of colonic lesions, this cross-sectional imaging technique offers an additional advantage over OC by enabling analysis of the entire abdominal–pelvic cavity and the lung bases. It can reveal benign, indeterminate or frankly pathological abnormalities, such as an abdominal aortic aneurysm or cancer. Within a screening population, the abnormality rate ranges from 4 to 16%, with 2–8% being frankly pathological [24]. The most frequently encountered extracolonic cancers include renal, lung and lymphoma [25].

Some authors have even proposed and evaluated the usefulness of CTC for opportunistic screening for cardiometabolic disorders related to chronic diseases, such as osteoporosis, sarcopenia and cardiovascular diseases [26]. Artificial intelligence algorithms enable automatic quantification of bone–mineral density, paravertebral muscle mass, visceral and subcutaneous fat, hepatic steatosis, and the aortic calcification score [27]. In contrast, some institutions [28, 29] consider that the detection of extracolonic lesions, particularly benign ones, during CTC can lead to unnecessary, costly and anxiety-inducing additional explorations for patients.

## Classification of CT findings

In 2005, the American College of Radiology (ACR) introduced the « CT Colonography Reporting and Data System (C-RADS) » to develop standardized terminology and report structure enabling a robust classification scheme for CTC findings [30]. Despite an update published in 2024 [31], this scheme used for colorectal and extracolonic findings at CTC remains not generally applied in European countries.

## CTC indications and non-indications

Despite its excellent performance, CTC remains underused for screening. However, numerous other indications supported by joint recommendations from ESGAR and the European Society of Gastrointestinal Endoscopy (ESGE) have expanded its application scope [10]. The reasons for CTC non-indications and contraindications are reported in Table 1 [32].

**Table 1:** Non-indications and contraindications of CTC

Context/situation	Indication
Individuals at very high risk of cancer, e.g., those carrying genes for familial adenomatous polyposis or hereditary non-polyposis colorectal cancer (Lynch syndrome)*	Not indicated
Routine follow-up inflammatory bowel disease	Not indicated
Evaluation of canal anal disease	Not indicated
Active inflammatory, infectious or ischemic colitis, or acute diverticulitis†	CTC contraindicated
Bowel obstruction	CTC contraindicated
Non-reducible inguinal hernia	CTC contraindicated
Recent polypectomy, mucosectomy, deep biopsy	CTC contraindicated
Existing or no excludable pregnancy	CTC contraindicated
Recent laparoscopy, laparotomy, subtotal colectomy	Not indicated
Children and adolescents	Not indicated

* These patients should be included in a specific screening program exclusively involving endoscopic examination

† For patients with acute diverticulitis, CTC can be scheduled 6–8 weeks after the acute phase

### Screening for colorectal cancer

With 1.8 million new cases per year worldwide, colorectal cancer ranks as the 4th most common cancer and is the second leading cause of cancer death [33]. Most organized colorectal cancer screening programs use noninvasive stool tests, such as immunochemical test or guaiac fecal-occult-blood test, whereas opportunist screening programs are based on OC. In the USA, the opportunistic approach consists of either a high-sensitivity stool-based test or a direct test such as CTC, which has been supported by the Centers for Medicare & Medicaid Services (CMS) since January 2025, from age 45 [34, 35].

An advanced adenoma ≥ 10 mm, which is a benign tumor precursor to cancer, is considered the ideal target for colorectal cancer screening [36]. Concerning the efficacy of detecting advanced adenomas ≥ 10 mm, CTC performs similarly to OC. CTC sensitivity and specificity for detection of those precursor lesions ranged, respectively, between 90% and 94%, and 86% and 96% [2, 37]. CTC sensitivity for the diagnosis of colorectal cancer is very high: 96% [8, 9]. In comparison, immunochemical stool testing used to identify large polyps or advanced adenomas has relatively low sensitivity (40%) [37].

The United States Preventive Services Task Force (USPSTF) recommends CTC as a first-line screening test, similar to OC, with the test to be repeated every 5 years if the initial test is negative [34]. European Society of Gastrointestinal Endoscopy (ESGE) and ESGAR guideline update 2020 recommended CTC as an option for CCR when there is no organized FIT-based population colorectal screening program and in case of positive FIT with incomplete or unfeasible OC [32]. However, CTC is rarely reimbursed for this indication.

### Incomplete OC

OC can be unfeasible or incomplete for variety of reasons related to patient or operator experience and this may occur more often than excepted (9–27%) [38].

The causes of OC failures are numerous: inadequate bowel preparation, tumoral or inflammatory stenosis, parietal hernia, dolichocolon, incomplete common mesentery and/or adhesions, particularly in women > 50 years with a history of pelvic surgery. Almost all colonic stenoses through which the endoscope cannot pass can be explored by CTC with a complete study of the upstream colon. Stenoses do not affect the quality of bowel preparation [39]. Caution is essential when CTC is requested as an immediate adjunct to colonoscopy, requiring confirmation from the OC operator that no polypectomy or mucosectomy procedures were performed.

## Elderly and/or frail patients

**Fig. 7:**
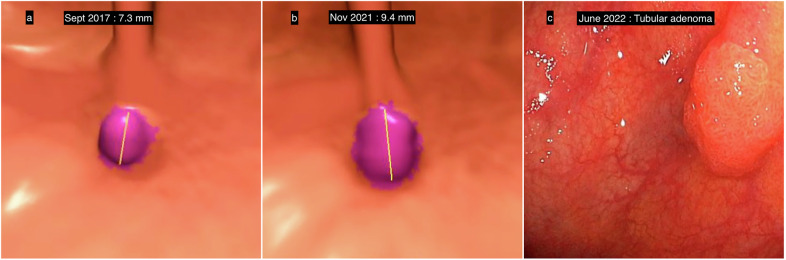
CTC surveillance of polyps in a 76-year-old female. **a** September 2017: CTC with a 3D endoscopic view showing a sessile polyp measuring 7.3 mm. The patient declined surgical intervention and requested surveillance. Follow-up CTC was proposed. **b** At the follow-up CTC, delayed 1 year by the COVID-19 pandemic, the polyp measured 9.4 mm, representing a 2.1-mm increase, i.e., 28.8% in this particular patient, with a 20% variation conventionally considered significant. **c** June 2022: optical colonoscopy prior to polypectomy, around 5 years after the initial colonoscopy. Histology diagnosed a tubular adenoma with low-grade dysplasia

Morphological exploration of the colon in elderly individuals is justified because their colorectal cancer incidence is higher than that of the general population. In this population, the percentage of incomplete OCs is higher due to poor adherence to bowel preparation, dolichocolons, adhesions and/or diverticular disease. The risk of adverse events during OC, like perforation or gastrointestinal bleeding, is higher for these patients often on anticoagulants. In addition, they have numerous contraindications to anesthesia, often rendering OC infeasible. Due to the advancing age of the population, CTC is becoming an essential minimally invasive, well-tolerated and safe triage approach, ensuring that only patients with positive findings are referred for OC. Patients with only subcentimeter polyps can benefit from CTC follow-up at 3 years (Fig. 7).

## Symptomatic patients

The onset of symptoms suggestive of colonic tumors, e.g., rectal bleeding, anemia and/or unexplained weight loss, necessitates morphological exploration of the colon. The results of the Special Interest Group in Gastrointestinal and Abdominal Radiology (SIGGAR) clinical trial showed that CTC and OC have similar performances in detecting colorectal cancer and tumors ≥ 10 mm in symptomatic individuals [5]. Moreover, CTC detection of 10% of extracolonic malignancies and 13% of extracolonic findings helped explain at least one of these patients’ symptoms. Symptoms suggestive of irritable bowel syndrome are not considered an indication for CTC, particularly in young patients [40].

## Suspected submucosal lesion during OC

CTC’s ability to visualize and thus analyze both sides of the colon wall and lumen provides useful information for characterizing OC-detected submucosal lesions. Submucosal lesions may correspond to tumors developing from the intestinal wall, like lipomas and mesenchymal tumors, or extramural lesions, like carcinomatous or endometriotic nodes. They may also correspond to pseudo-lesions caused by extrinsic impressions from neighboring organs, or colonic pneumatosis.

**Fig. 8:**
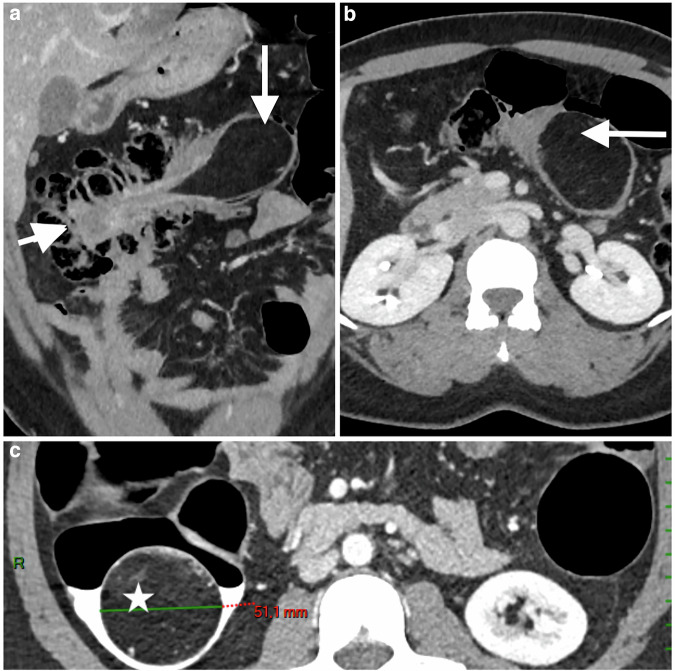
Giant lipoma of the right colon (white arrow). **a** Axial and **b** coronal abdominal CT-scan images, showing a pedunculated colonic lipoma causing upstream colocolic intussusception (arrowhead), resulting in an erroneous location on the left colon. **c** Axial CTC image after CO_2_ insufflation reverse-telescoped the right colon and precisely located the giant 50-mm lipoma (white star) within it

Lipomas are the most common submucosal tumors. Although their diagnosis is usually straightforward, it can sometimes be misleading, particularly for giant lipomas (> 2 cm) responsible for colocolic intussusception that may sometimes impede the progression of the endoscope (Fig. 8). CTC provides a definitive diagnosis of lipomas by measuring the characteristic fat density, specifying its location and searching for synchronous lesions in the case of obstructive tumors. These comprehensive findings help surgeons plan the surgical procedure. For endometriosis with digestive involvement, CTC complements the conventional preoperative assessment, which includes endovaginal ultrasound and pelvic MRI [41].

## Diverticular disease of the sigmoid colon

**Fig. 9:**
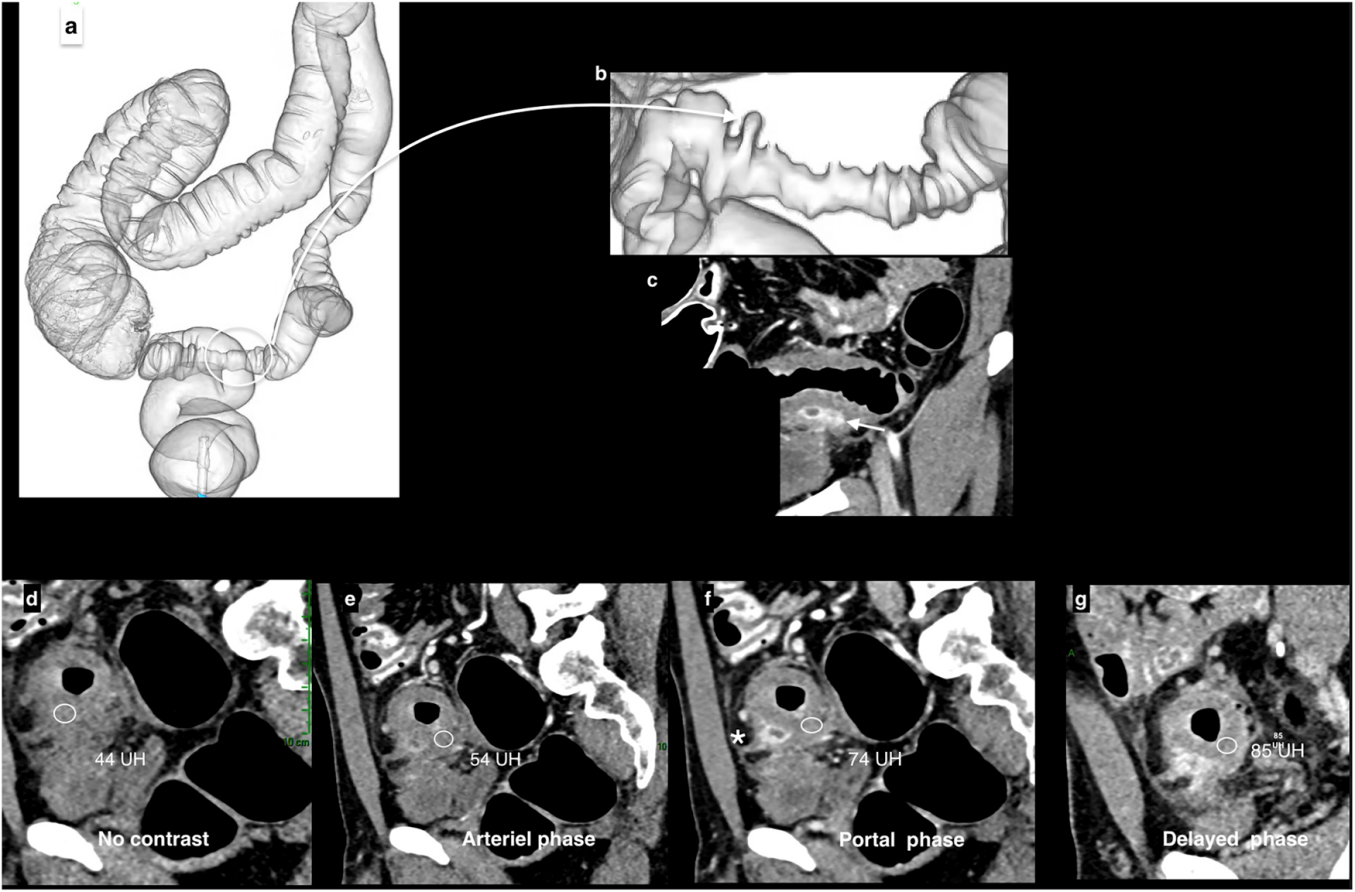
Morphological follow-up evaluation, 4 months after acute sigmoid diverticulitis, of a 35-year-old patient with a sigmoid stenosis and incomplete optical colonoscopy. **a** CTC provided a complete overview of the colon, ruling out the presence of an underlying neoplasm and/or another inflammatory colon disease, such as chronic inflammatory bowel disease. **b** Enlargement of the zone encircled in (**a**) showing tight, centered and symmetrical sigmoid stenosis with diverticulosis. **c** The 2D-coronal view shows persistent signs of disease activity with small abscesses (despite a 4-month delay after the acute episode) (white arrow) within the chronic thickened digestive wall. **d**–**g** Sagittal view perpendicular to the long axis of the inflammatory sigmoid: the delayed intravenous contrast-medium enhancement of the colon wall suggests parietal fibrosis. **f** Note the linear fistula between the abscesses and the peritoneum, highlighted by contrast-medium uptake, seen during the portal phase (asterisk)

Acute diverticulitis is typically diagnosed with an abdominal–pelvic CT scan. When diverticular sigmoiditis is complicated by an abscess or enteral fistula, the preoperative assessment, conducted sometime after the acute episode, includes OC to exclude a sigmoid colon cancer mimicking complicated diverticulitis and to explore the proximal colon. However, when OC is incomplete or contraindicated, CTC is preferably scheduled 6–8 weeks after the acute episode. CTC evaluates the extent of the diverticular disease throughout the colon and determines the severity score of diverticular disease (SSDD) on a scale of 1 to 4 proposed by Flor et al [15]. The SSDD is based on intestinal wall-thickening and the diminished intestinal lumen diameter (Table 2). The highest score corresponds to the presence of established and irreversible fibrosis. This classification helps select patients who are candidates for preventive sigmoidectomy (Fig. 9).

**Table 2:** Diverticular disease severity score (DDSS) [13]

Score	Maximum colon-wall thickness	Minimum intestinal lumen diameter
1	< 3 mm	≥ 15 mm
2	3–8 mm	≥ 5 mm
3	≥ 8 mm	≥ 5 mm
4	≥ 8 mm	< 5 mm

The degree of severity of diverticular disease using the DDSS score based on maximum sigmoid colon wall thickness and minimum lumen diameter from inner-to-inner wall

## Colon-cancer management

**Fig. 10:**
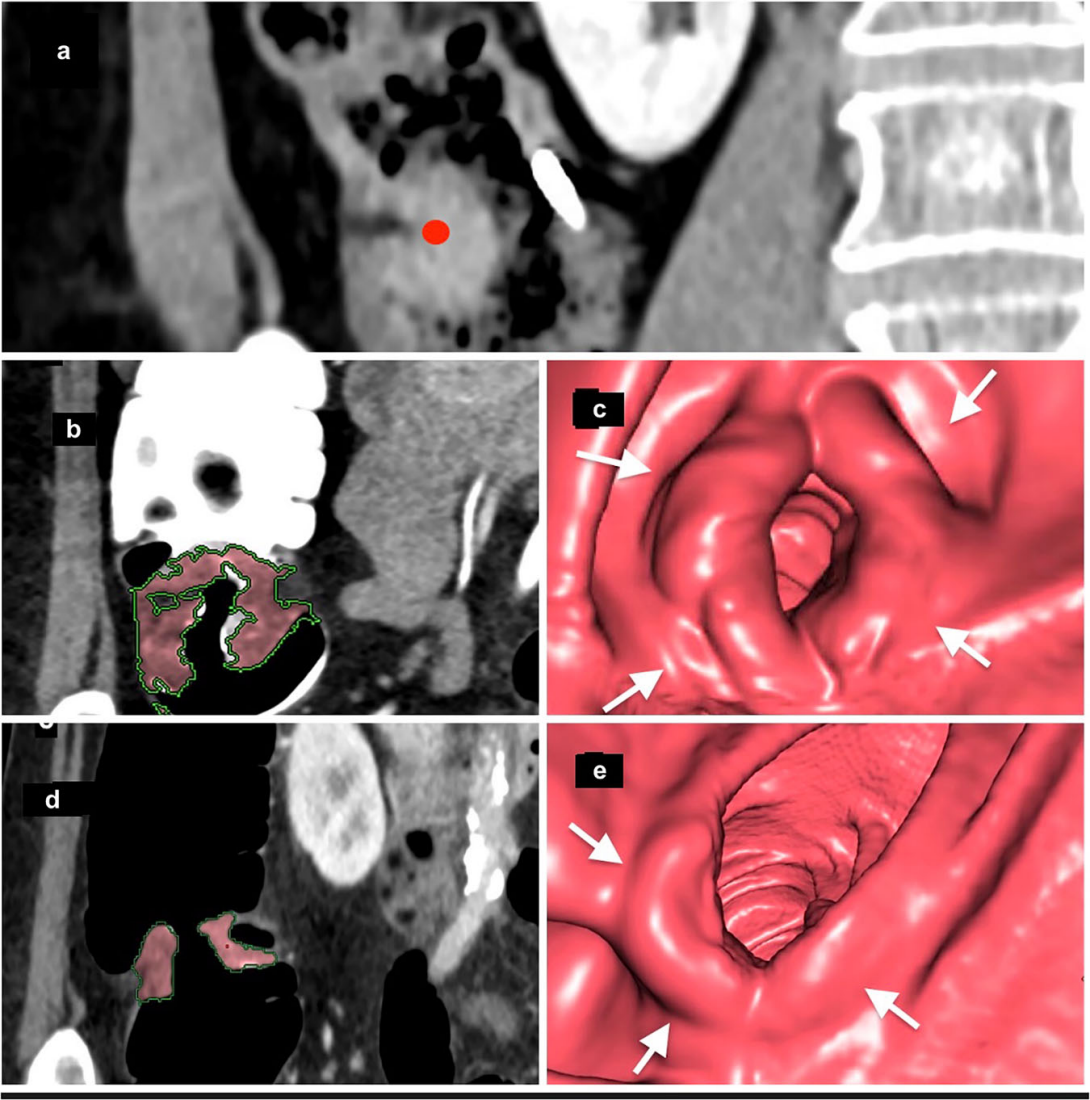
Advanced local colonic tumor response after neoadjuvant immunotherapy (NAI). **a** CT abdominal scan after optical colonoscopy: soft-tissue lesion (red dot) close to the clip. The margin tumor is imprecise and difficult to measure. **b**, **d** 2D-coronal views show the tumor volume calculated from semi-automatically selected voxels. The tumor volume was precisely 16.8 cm^3^ (**c**) before and 7 cm^3^ after NAI (**e**). **c**, **e** The 3D-endoscopic views enable assessment of the tumor response: the tumor occupies **c** the total circumference (white arrows) before NAI and **e** only half of the circumference (white arrows) post-NAI

Surgical treatment of colon cancer has evolved significantly with laparoscopy, which has now become the reference technique for colectomies. Study results have demonstrated the effectiveness of neoadjuvant immunotherapy for colon cancer [42, 43]. To meet these new needs of therapists, CTC is gaining increasing interest (Fig. 10).

### Preoperative assessment

The classic preoperative assessment for colon cancer includes OC and contrast-enhanced TAP-CT. While OC’s role is most often to guide the biopsy of the primary neoplasm, CT has traditionally been used to detect metastases. However, OC is limited to exploration of the proximal colon by an obstructive tumor and might imprecisely specify the tumor’s location, especially in cases of dolichocolon. TAP-CT assesses metastases but does not always detect the primary tumor (e.g., small or flat tumor) and may underevaluate the local tumor-staging assessment [44].

Accurate assessment of the tumor’s location is useful for preoperative planning and crucial during the surgical procedure, especially laparoscopy, as the field of vision is limited and digital palpation is impossible. CTC always determines with greater precision the colon tumor site, thereby improving the surgeon’s confidence in planning and performing the operation [14, 45, 46].

Occlusive colon cancer prevents OC exploration of the proximal colon. Moreover, since most of these tumors predominate in the left colon and sigmoid, most of the colon remains unexplored. Colorectal cancer patients who undergo complete preoperative colon evaluation experience fewer local recurrences, have a lower risk of developing metastases and ultimately survive longer than patients with incomplete OC [47].

**Fig. 11:**
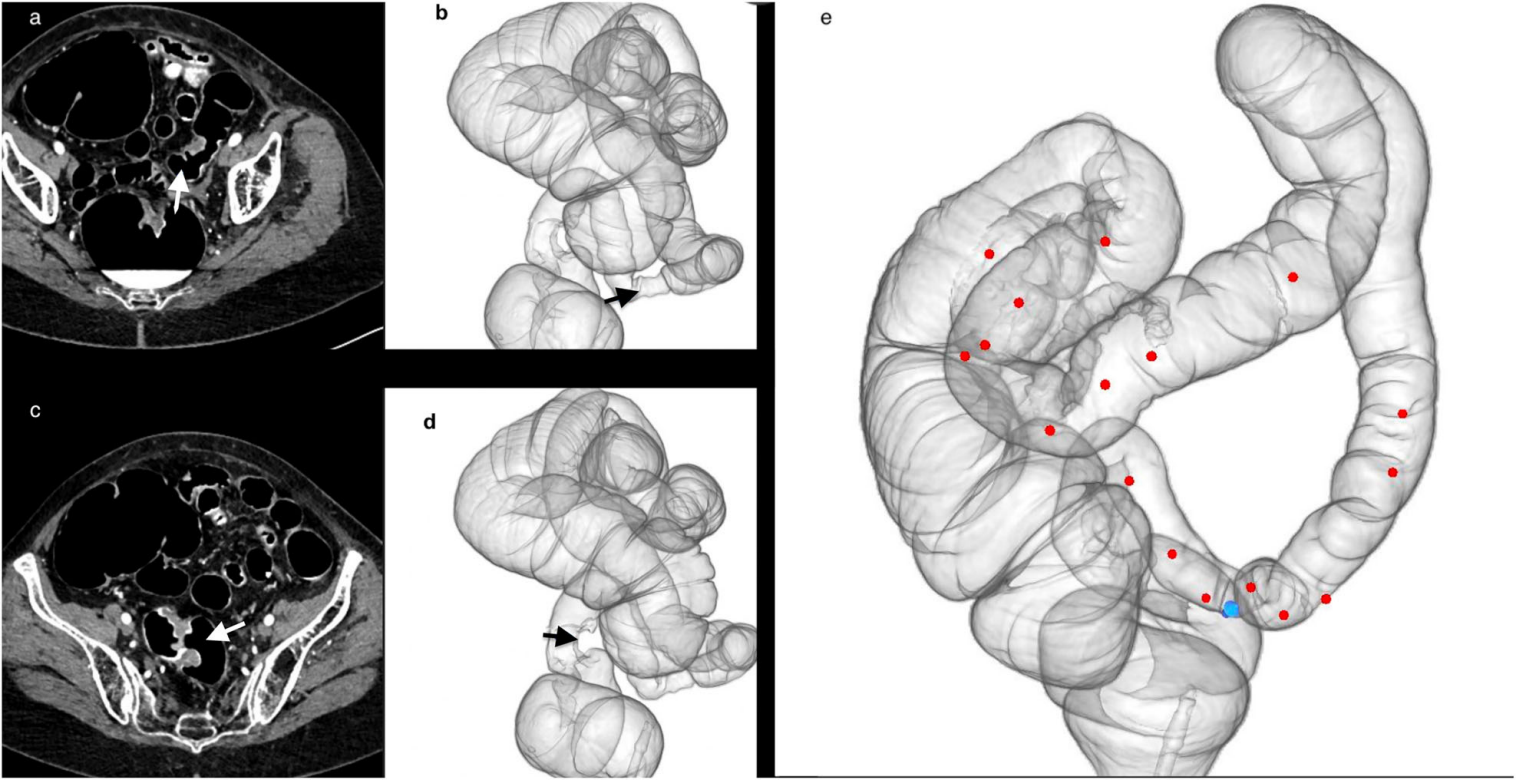
Preoperative planning for ablation of synchronous cancers and polyps. The patient was referred for an incomplete optical colonoscopy (OC), which revealed the presence of two colorectal cancers in the sigmoid region and several polyps in the left colon, which could only be partially explored. CTC enabled complete exploration of the colon, identifying the two cancers seen on the 2D axial images located **a** in the sigmoid itself (white arrow) and **c** at the rectosigmoid junction (white arrow). **b**, **d**, **e** 3D overviews of the colon indicate the precise locations of the two synchronous cancers (black arrows), and **e** the precise repartition of intermediate (6–9 mm) polyps (red dots) and their numbers, details not completely provided by OC. Note the blue bookmark in the sigmoid. Initial planning foresaw subtotal colectomy. CTC led to plan modification to a complete laparoscopic left colectomy, followed by therapeutic OC left colectomy within 6 months

CTC has demonstrated that the synchronous cancer frequency is 4–6% [48]. Synchronous colon cancer upstream from the obstructive tumor changes the surgical planning for 7% of patients [16, 48, 49] (Fig. 11). CTC detection of a synchronous polyp > 10-mm upstream from the obstructive tumor will trigger postoperative therapeutic OC with polypectomy.

**Fig. 12:**
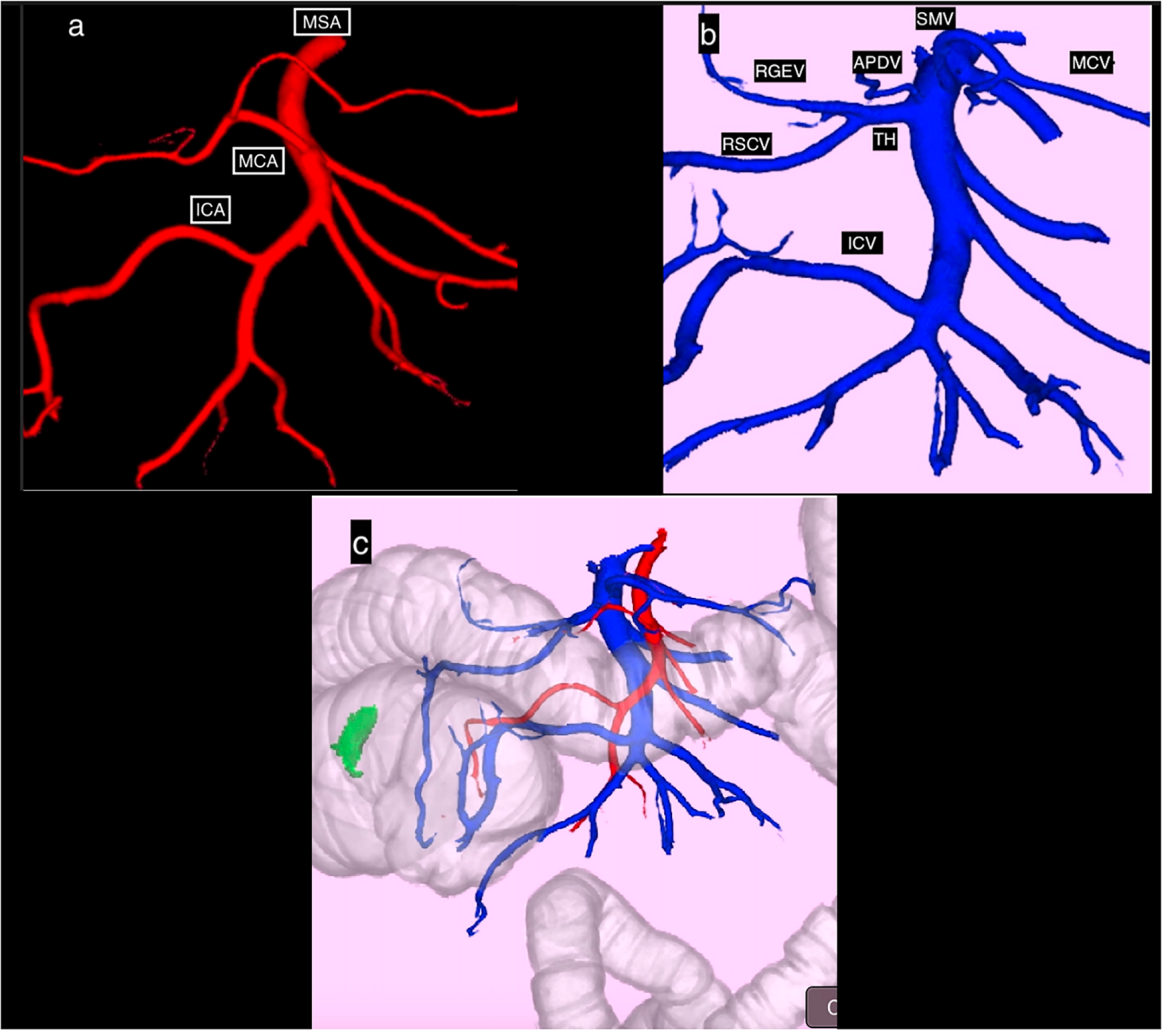
3D-CTC-angiography (CTC-A). **a** Map of the mesenteric colic artery (MSA), middle colic artery (MCA) and ileocolic artery (ICA). The right colic artery is absent. **b** Map of the superior mesenteric vein (SMV) and its confluent branches. The gastrocolic trunk, also known as the Henle trunk, is formed by the confluence of the right gastroepiploic vein (RGEV) and the right superior colic vein (RSCV). The anterior pancreaticoduodenal vein (APDV) and the middle colic vein (MCV) join the SMV separately, as does the ileocolic vein (ICV). **c** Overall view of the global colon morphology, with the precise location of the tumor (in green) and mesenteric vascular mapping, making it possible to determine the positions of the colonic SMA and SMV branches

Mesenteric vascular, arterial and venous mapping is essential for preoperative planning of laparoscopy. Identifying anatomical vascular variants lowers the intraoperative hemorrhagic risk and optimizes lymph node dissection. Coupling CTC and CT-angiography of the abdominal aorta obtains 3D-CTC-A which provides in a single examination, local tumor-extension assessment by determining its precise location and establishing a mesenteric vascular map at the same time (Fig. 12).

### Postoperative surveillance

When the preoperative OC was complete and of good quality, postoperative colon cancer surveillance includes a TAP-CT and OC at 1 year to search for metastases, metachronous cancer and/or anastomotic recurrence.

Weinberg et al compared CTC performances with contrast-enhanced TAP-CT and patient preference (*n* = 231 patients) to the classical combination of TAP-CT and OC [50, 51]. CTC detected extra-luminal and perianastomotic recurrences, which were inaccessible by OC and non-metachronous tumors. However, sensitivity for polyps ≥ 5 mm was 44%, and only 22% of patients preferred CTC. Therefore, ESGE-ESGAR recommends CTC only in these patients when OC is contraindicated or unfeasible.”

For patients undergoing emergency surgery for occlusion with a diverting colostomy, exploration of the upstream colon is necessary before restoring continuity. CTC exploration of the proximal colon via the colostomy orifice performs equally as well as OC and with greater comfort for the patient [38].

## CTC risks and complications

The X-ray exposure for CTC is 1–3 mSv every 3–5 years, depending on the surveillance frequency, compared to natural radiation of 4.5 mSv per year [52]. This low dose has been achieved by technological advancements in detectors and iterative reconstruction filters incorporating artificial intelligence.

Colorectal perforation during CTC is an exceptional complication. In a Japanese national survey, Nagata et al found the colorectal perforation rate during preoperative-staging CTC was 0.028%, while the perforation rates for screening and diagnosis were 0.003% and 0.014%, respectively [53]. In a meta-analysis from Bellini et al based on 100,000 patients, the colorectal perforation rate after a CTC was 0.04% and only 0.02% in asymptomatic subjects, with an induced surgery rate of 0.008% [54].

The ability to use pressure and volume measurements as surrogate measures to determine distention adequacy to begin scanning leads to secure and highly reproducible colon distention. CTC is a minimally invasive and safe examination for patients when its contraindications are respected (Table 1).

## Training radiologists

Identification of a colonic neoplasm during a follow-up examination raises questions about the normality of the previous examination results (so-called interval cancer). This post-imaging colon cancer rate is an objective evaluation of radiologist performance as well as the post-OC colorectal cancer rate for the endoscopist. A review of the recent literature has shown that this rate is 4% over 3 years for CTC, which is close to that of OC rates ranging from 2.9 to 8.6% [55]. More than half of cancers diagnosed after normal CTC are retrospectively visible on reviewed CTC images and are due to perceptual errors [55].

That CTC performance has been demonstrated to be equivalent to that of OC holds true only when the examination is performed according to best practices and interpreted by trained radiologists. Those two conditions are essential to avoid misinterpretations that could discredit the method. Results of numerous studies indicate that insufficient mastery of specific CTC evaluation techniques leads to detection and perceptual errors, resulting in false negatives (missed lesions) and false positives, prompting unwarranted OCs [55, 56]. Despite specific training, low performance may be attributable to the absence of a post-training test that would have selected only those radiologists who have reached the performance level required to participate in the study. That situation emphasizes the need for a well-developed, structured training program with clear objectives and a quality-control strategy, including accreditation similar to breast cancer screening with mammography. Specific training programs have long been implemented in gastroenterology for the practice of OC screening. Various CTC-training programs have been offered by professional societies worldwide, including the ESGAR. These programs include a panel of both normal and pathological examinations, all proven by endoscopy. The number of cases varies between 50 and 175 cases with an aspirational target of 300 cases for the British Society of Gastrointestinal and Abdominal Radiology and the Royal College of Radiologists (BSGAR-RCR) [57, 58].

The American College of Radiology recommends that CTC specialists complete 50 endoscopy-verified cases every 2 years as continuing education. Double readings or centralized reading by experts is another potential strategy to maintain this performance level. In addition, radiologists should not be obliged to manage too many CTCs in a single day or pressured to analyze images too quickly [59].

## Conclusion

Even though extensive research confirms the outstanding diagnostic accuracy of CTC in detecting polyps ≥ 10 mm and colorectal cancer, CTC remains underused. Once viewed as a competitor to OC, CTC now occupies a distinct niche in colonic lesion evaluation, embodying a complementary approach, in which each technique excels in areas where the other may falter. In many centers, CTC is not recommended as a primary screening tool for colorectal cancer but serves as a valuable adjunct for monitoring inconclusive or equivocal OC findings. It offers a viable alternative to OC for elderly and/or debilitated patients and those averse to undergoing OC under general anesthesia.

CTC’s ability to provide precise staging of locally advanced tumors, guiding decisions on neoadjuvant immunotherapy, underscores its potential usefulness in this setting. Coupled with contrast-enhanced TAP-CT, CTC enables comprehensive, preoperative evaluation for laparoscopic colectomy and staging within a single diagnostic session. Furthermore, CTC’s capability to detect clinically significant extracolonic abnormalities and facilitate opportunistic screening for cardiometabolic conditions, precursors to chronic illnesses, highlights its broader impact beyond colorectal disease.

## Abbreviations

CTC: Computed tomography colonography
CTC-A: CTC–angiography
ESGAR: European Society of Gastrointestinal and Abdominal Radiology
FIT: Fecal immunochemical test
LST: Lateral spreading tumor
LST-G: LST granular
LST-NG: LST non-granular
OC: Optical colonoscopy
SSDD: Severity score of diverticular disease
TAP-CT: Thoracic–abdominal–pelvic CT
